# Bioanalytical screening of low levels of dioxins and dioxin-like PCBs in pig meat (pork) for checking compliance with EU maximum and action levels using highly sensitive “third generation” recombinant H4L7.5c2 rat hepatoma cells

**DOI:** 10.1186/s12302-021-00474-2

**Published:** 2021-03-18

**Authors:** Johannes Haedrich, Claudia Stumpf, Michael S. Denison

**Affiliations:** 1 European Union Reference Laboratory (EU-RL) for Dioxins and PCBs in Feed and Food, CVUA Freiburg, Bissierstraße 5, 79114 Freiburg, Germany; 2 Department of Environmental Toxicology, University of California Davis (UCD), One Shields Avenue, Davis, CA 95616 USA; 3 Ringstr. 5, 79252 Stegen, Germany

**Keywords:** Bioanalytical screening, CALUX bioassay, Dioxins, Dioxin-like PCBs, Pig meat, EU Legislation

## Abstract

**Background:**

Low maximum and action levels set by the European Union for polychlorinated dibenzo-p-dioxins and dibenzofurans (PCDD/Fs) and dioxin-like polychlorinated biphenyls (DL-PCBs) in pig meat (pork) have led to a demand for reliable and cost-effective bioanalytical screening methods implemented upstream of gas chromatography/high-resolution mass spectrometry confirmatory technology, that can detect low levels of contamination in EU-regulated foods with quick turn-around times.

**Results:**

Based on the Chemically Activated LUciferase gene eXpression (CALUX) bioassay, extraction and clean-up steps were optimized for recovery and reproducibility within working ranges significantly lower than in current bioassays. A highly sensitive “3rd generation” recombinant rat hepatoma cell line (H4L7.5c2) containing 20 dioxin responsive elements was exposed to pork sample extracts, and their PCDD/Fs and DL-PCBs levels were evaluated by measuring luciferase activity. The method was validated according to the provisions of Commission Regulation (EU) 2017/644 of 5 April 2017 with spiking experiments performed selectively for PCDD/Fs and DL-PCBs and individual calibration for PCDD/Fs, DL-PCBs and the calculated sum of PCDD/Fs and DL-PCBs. The resulting performance parameters met all legal specifications as confirmed by re-calibration using authentic samples. Cut-off concentrations for assessing compliance with low maximum levels and action levels set for PCDD/Fs and DL-PCBs within a range of 0.50–1.25 pg WHO-TEQ/g fat were derived, ensuring low rates of false-compliant results (ß-error < 1%) and keeping the rate of false-noncompliant results well under control (α-error < 12%).

**Conclusions:**

We present a fast and efficient bioanalytical routine method validated according to the European Union’s legal requirements on the basis of authentic samples, allowing the analyst to reliably identify pork samples and any other EU-regulated foods of animal origin suspected to be noncompliant with a high level of performance and turn-around times of 52 h. This was facilitated in particular by a quick and efficient extraction step followed by selective clean-up, use of a highly sensitive “3rd generation” H4L7.5c2 recombinant rat hepatoma cell CALUX bioassay, and optimized assay performance with improved calibrator precision and reduced lack-of-fit errors. New restrictions are proposed for the calibrator bias and the unspecific background contribution to reportable results. The procedure can utilize comparably small sample amounts and allows an annual throughput of 840–1000 samples per lab technician. The described bioanalytical method contributes to the European Commission's objective of generating accurate and reproducible analytical results according to Commission Regulation (EU) 2017/644 across the European Union.

## Introduction

The collective term “dioxin” covers a total of 75 polychlorinated dibenzo-p-dioxins (PCDDs) and 135 dibenzofurans (PCDFs), and generally refers to those specific congeners that produce a common spectrum of toxic and biological effects [1–3]. The prototypical and most potent/toxic member of this class of compounds is 2,3,7,8-tetrachlorodibenzo-p-dioxin (2,3,7,8-TCDD). In general, dioxins are formed during combustion processes, when organic compounds oxidize in the presence of halogen compounds, especially chlorine or bromine. This is the case with smelting, waste incineration, chlorine bleaching of paper pulp, and the production of some herbicides and pesticides, but also with natural processes such as forest fires or thunderstorms. These toxic dioxin-like chemicals are frequently highly lipophilic and resistant to metabolic degradation and as such, once these compounds enter the body, they are difficult to eliminate, with a 2,3,7,8-TCDD half-life in adult humans ranging from 3 to 10 years [4]. Body burdens of PCDD/Fs in humans mainly depend on age, eating habits, and weight changes, as well as on breastfeeding in women and on the breastfeeding period in infants and young children. In addition, some of the structurally related 209 polychlorinated biphenyls (PCBs) also produce dioxin-like biological and toxicological effects, and are referred to as dioxin-like PCBs (DL-PCBs).

The European Union (EU)-regulated, toxicologically most relevant dioxin-like compounds include 17 structurally related PCDD/Fs and 12 DL-PCBs which are often detectable in a variety of foods. Levels of dioxins and DL-PCBs are normally expressed in TEQs (Toxic EQuivalents). The concentrations of the different toxic congeners are multiplied by a toxic equivalency factor (TEF), expressing their toxicity relative to the most toxic congener, 2,3,7,8-TCDD, and the results summed up to generate an overall TEQ for the sample [5, 6]:

$${\text{TEQ}} = \sum\limits_{i = 1}^{7} {\left( {{\text{PCDD}}_{i} \cdot {\text{TEF}}_{i} } \right) + \sum\limits_{j = 1}^{10} {\left( {{\text{PCDF}}_{j} \cdot {\text{TEF}}_{j} } \right) + \sum\limits_{k = 1}^{12} {\left( {{\text{DL - PCB}}_{k} \cdot {\text{TEF}}_{k} } \right)} } } .$$

The primary exposure source for polychlorinated dioxins in humans (> 90%) is via consumption. According to the European Food Safety Authority (EFSA), main contributors are “fatty fish” (up to 56% contribution), “unspecified fish meat” (up to 53.4% contribution), “cheese” (up to 21.8% contribution), and “livestock meat” (up to 33.8% contribution) [7].

Based on new epidemiological and experimental data on the toxicity of these substances and more refined modelling techniques for predicting human body levels over time, the EFSA Panel on Contaminants in the Food Chain (CONTAM) established in 2018 a new tolerable weekly intake (TWI) for dioxins and DL-PCBs in food of 2 pg TEQ per kg of body weight (bw) [7]. This new TWI is seven times lower than the previous EU tolerable intake set by the European Commission’s former Scientific Committee on Food in 2001 [8]. Within European countries, however, the mean and P95 intake of total TEQ by adolescents, adults, elderly, and very elderly varies between, respectively, 2.1–10.5, and 5.3–30.4 pg TEQ/kg bw/week, implying a considerable exceedance of the TWI [7].

The EU has in Commission Regulation (EC) No 1881/2006 of 19 December 2006 [9], last amended by Commission Regulation (EC) No 1067/2013 of 30 October 2013 [10], established maximum levels (MLs) for PCDD/Fs and for the sum of PCDD/Fs and DL-PCBs in certain foodstuffs to protect public health by reducing the presence of dioxins and PCBs in the environment, feed, and food. In addition to the legally binding MLs, the European Commission endorsed in Recommendation 2011/516/EU of 23 August 2011 [11], the use of voluntary action levels (ALs) set around 2/3 of the ML. ALs are intended to serve as early warning tools for authorities and operators to highlight cases where it is appropriate to identify a source of contamination and to take measures for its reduction or elimination. The most recent set of ALs for dioxins and PCBs in food is given in Commission Recommendation 2014/663/EU of 11 September 2014 [12].

Gas chromatography coupled to high-resolution mass spectrometry (GC/HRMS) is applied whenever individual congeners of PCDD/Fs and DL-PCBs need to be identified and quantified, e.g., in cases when results from previous analyses must be confirmed or when congener (source) patterns (“fingerprints”) need to be established. For routine screening, however, complementary high-throughput, easy-to-run, and cost-effective cell-based bioanalytical methods have more recently emerged and are now well established in a number of laboratories across Europe and also world-wide. These “bioassays” are implemented upstream of GC/HRMS technology, reliably monitoring concentrations and identifying samples suspected to be noncompliant with the respective legal limits and have relatively short turn-around times. Analytical criteria and requirements [13] for validation, run acceptance, and quality control based on results from confirmatory methods such as GC/HRMS were adopted by EU legislation in 2012 [14, 15], amended in 2014 [16, 17], and again in 2016 [18, 19] for use in official feed and food control.

Within the scope of establishing strong EU-wide standards for routine and reference methods, the Bioassay Research Unit at the *European Union Reference Laboratory (EU-RL) for Dioxins and PCBs in Feed and Food* (recently named the “EU-RL for Halogenated Persistent Organic Pollutants in Feed and Food”) has evaluated and optimized the performance of the Chemically Activated LUciferase gene eXpression (CALUX) bioassay with a focus on its use within European official feed and food control [20–22]. CALUX detects 2,3,7,8-TCDD and structurally related halogenated aromatic hydrocarbons (HAHs) based on their ability to activate the aryl hydrocarbon receptor (AhR) signalling pathway [20, 23] and was first described by Denison and co-workers [24–27].

Correspondence of bioanalytical results expressed as Bioanalytical EQuivalents (BEQs) with results from confirmatory instrumental methods expressed as TEQs, in which EU regulatory limits are given, is an essential outcome of validation and quality control QC procedures. BEQ/TEQ ratios must be evaluated by calibration studies for those EU-regulated sample matrices or matrix groups to which MLs and/or ALs were assigned. BEQ-based matrix-dependent cut-off concentrations ensuring a false-compliant rate (ß-error) < 5% shall be established, above which a sample is declared suspected to exceed the respective legal limit, requiring follow-up by confirmatory analysis. This concept requires close co-operation between the two partner-labs and may, by sieving out most of the compliant samples, considerably reduce the workload of the lab running the confirmatory method.

Bioanalytical methods for separate analysis of PCDD/Fs and DL-PCBs, and of the sum of PCDD/Fs and DL-PCBs in 20 EU-regulated food matrices were validated by the *EU-RL Bioassay Research Unit* [28–31]. Method performance was demonstrated for each matrix in a range between “0” and 2xML, for the respective MLs and ALs. MLs (and consecutively, ALs), however, were not established on a safety-based approach but using the principle of “strict but feasible” [32], by setting these limit values based on data obtained from EU member states around the 90th-to-95th percentile of the distributions of contaminant levels in food (and feed) produced using good agricultural practices (GAP). This led to relatively low MLs [33] and ALs [12] for dioxins and dioxin-like PCBs in *pig meat* (pork) and products thereof [20]:

$$\begin{array}{*{20}l}{\text{Maximum levels:}} & \\ {{1}.{\text{25 pg WHO}} - {\text{PCDD}}/{\text{F}} - {\text{PCB}} - {\text{TEQ}}/{\text{g fat}}} & \\ {{1}.0{\text{ pg WHO}} - {\text{PCDD}}/{\text{F}} - {\text{ TEQ}}/{\text{g fat}}} \end{array} $$

$$\begin{array}{*{20}l}{{\text{Action levels:}}} &\\ {0.{\text{75 pg WHO}} - {\text{PCDD}}/{\text{F}} - {\text{TEQ}}/{\text{g fat}},} &\\{0.{5}0{\text{ pg WHO}} - {\text{PCB}} - {\text{TEQ}}/{\text{g fat}}} \\ \end{array}$$

This paper outlines results from development, optimization, validation, and routine quality control of the bioanalytical screening of pig meat (pork) samples with low levels of contamination, for checking of sample compliance with low legal limits while generating analytical results “close” to the previously customary lower ends of assay and method working ranges.

## Methods

### Chemicals and glassware

Acetone, cyclohexane, ethyl acetate, n-hexane, propan-2-ol, and toluene, each of grade “Dioxins, Pesti-S, Furans, PCBs analysis” were from Biosolve (Valkenswaard, The Netherlands). Every new batch of organic solvent was pre-tested in our laboratory for the presence of AhR-active compounds using our most sensitive cell lines, following strict protocols. Double-distilled water (ddH_2_O) was from Roth (Karlsruhe, Germany), and DMSO (99.9%, for spectroscopy, Acros Organics™) and EDTA (99 + %, for analysis, Acros Organics™) from Fisher Scientific (Schwerte, Germany). Activated charcoal for determination of AOX, Celite 545 (particle size 0.02–0.1 mm), Silica gel 60, and sulfuric acid (95–97%) were from Merck (Darmstadt, Germany). Sodium sulfate (anhydrous, granular, 12–60 mesh) was from Baker (Phillipsburg, NJ, USA), and prior to use, it was heated at 450 °C for 10 h and allowed to cool to ambient temperature in a desiccator. Trypsin (2.5%) in balanced salt solution (BSS), cell culture medium (α-MEM, cat. no. 22561-054), and fetal bovine serum (FBS, EU Approved, South American, cat. no. 10270-106) were obtained from Gibco, Carlsbad, CA, USA.

Cleaned standard laboratory glassware was baked at 435 °C for 18 h (overnight), and after cooling to ambient temperature, the glassware was kept covered or stored in a suitable container. Shortly before use, the glassware was rinsed three times with n-hexane and briefly allowed to dry. All glassware was loosely covered with aluminum foil throughout sample processing.

### Sample material

Pork samples were obtained during official routine control programs from regular market food and from contamination incidents and collected over a period of 2 years. Each sample consisting of 2 kg each was thoroughly homogenized and, if not immediately analyzed, stored below − 25 °C until analysis.

### Sample extraction and clean-up

Following a fast and efficient modification of Smedes’ method [34], “total” lipids were extracted from each sample within just 6 min. Details of the procedure, which has been demonstrated to be applicable to each EU-regulated sample of animal origin, were published elsewhere [35].

In principle, aliquots of 2-propanol, cyclohexane, and ddH_2_O in a ratio of 8:10:8 (v/v/v) were added to 5 g of homogenized pig meat weighed in a Duran glass bottle (250 mL, Schott, Mainz, Germany). The analytical sample weight was chosen according to expected lipid contents and concentrations of contaminants to be quantified. The mixture was dispersed with an Ultra-Turrax® disperser (IKA T25, IKA, Staufen, Germany) with an 18 mm dispersing element (IKA S25N-18G) at 10,000 rpm for 30 s. Spontaneous phase separation occurred within 30 s. The upper layer was transferred into an evaporation glass tube using an accu-jet® pro pipette controller with adjustable speed (Brand, Wertheim, Germany) equipped with a glass pipette. Extraction was repeated twice with additional cyclohexane and 15 s dispersing time. The combined organic layers were reduced to dryness in a TurboVap II concentrator workstation (Biotage, Uppsala, Sweden) under mild conditions (water bath: 45–50 °C, nitrogen: 0.6 bar) to minimize evaporation losses particularly of the semi-volatile dioxin-like coplanar PCB 126, until the weight remained constant (in this study, approximately 0.9 g or 18% of extracted lipids were found).

In comparison to keeping the sample material under reflux with a suitable solvent (mixture) for a period of 4 h or longer on a Twisselmann extractor, a method widely used in traditional dioxin analysis, our rapid extraction yielded 99–105% of extractable lipids, more than 99.9% of which were re-dissolvable, e.g., in n-hexane [35]. Moreover, the short contact times between solvents and sample surface may largely prevent co-extraction of compounds structurally similar to the target analytes or activating the AhR signaling pathway for any other reason. Together with various other features, this method significantly improves the selectivity of the assay [35].

After re-dissolving the dry extract in 15 mL n-hexane, the solution was purified for screening analysis of PCDD/Fs and DL-PCBs by removing the lipids and any undesired AhR-active compounds that may cause false-noncompliant results, or compounds that may decrease or suppress the response (e.g., AhR antagonists/inhibitors) [21], using 33% sulfuric acid-activated silica (1:2, w/w). This step was followed by fractional elution of the target compounds from activated carbon/celite (1:99, w/w), using a mixture of n-hexane/toluene/ethyl acetate (8:1:1 v/v/v) for PCDD/Fs and toluene for DL-PCBs.

Carbon and celite were pre-tested, then mixed in a powder mixer via a number of “dilution” steps to yield a homogeneous mixture. Instead of introducing undesired AhR-active compounds into the final extract as had repeatedly been assumed, the carbon purifies both the extract and the applied solvents even further, eventually leading to sufficiently low procedural (reagent) blank values in the assay. According to legal requirements, the latter must be smaller than one-third of the sample concentration corresponding to the respective ML or AL [18, 19, 22]. The volumes of PCDD/F and DL-PCB eluates were reduced to 0.5 mL, transferred to a conical 1.2 mL vial, and carefully reduced further to 2–3 µL, followed by exchange of the solvent for 7 µL of DMSO in the case of the PCB-fraction, and for 14 µL of DMSO in the case of the dioxin/furan-fraction, following a well-established procedure, which keeps vial-to-vial variability, expressed as relative standard deviation (RSD), below 5%. The smaller volume for the final extract containing DL-PCBs was chosen due to a reduced relative potency (REP) or response of the cell bioassay to PCB 126, the most abundant DL-PCB, which is approximately 40% compared to the response of the assay to the most potent compound, 2,3,7,8-TCDD [21, 35–38]. The small final extract volumes provide concentration factors of, roughly, 700 for DL-PCBs and 350 for PCDD/Fs, the magnitude of which may pose a challenge to the control of unwanted and potentially interfering co-extractives or co-eluents altering the cell response.

### Chemically activated luciferase gene expression (CALUX) assay

A “3rd generation” H4L7.5c2 recombinant rat hepatoma CALUX cell line [39] that contains a stably transfected AhR-responsive firefly luciferase reporter gene under control of 20 dioxin responsive elements (DREs) [40] was utilized in this study. These cells are significantly more sensitive and responsive than previously reported CALUX cell lines given the amplification of the number of AhR DNA-binding sites (i.e., DREs) [20, 23, 41]. H4L7.5c2 rat and other CALUX cell lines, such as H4L1.1c4 rat and H1L6.1c3 mouse hepatoma cells (each with 4 DREs), or “3rd generation” H1L7.5c3 mouse hepatoma cells (20 DREs), are freely available for non-profit research purposes and can be obtained from Prof. Denison. These cells are also available for commercial and government screening purposes through a licensing agreement with the Hiyoshi Corporation, Omihachiman, Japan (www.calux-jp.com/english/).

Exposure of H4L7.5c2 rat cells to dioxins, dioxin-like compounds, and other AhR agonists results in induction of luciferase reporter gene expression in a time-, concentration-, AhR-, and chemical-specific manner [20]. The AhR signal transduction pathway in the CALUX bioassay and the molecular mechanism of activation of gene expression by 2,3,7,8-TCDD and related AhR agonists have been described in detail elsewhere [3, 20, 23, 26]. The level of luciferase expression in CALUX cell lines is directly related to the overall concentration of added AhR activators [23, 26].

### Measurement of luciferase activity and evaluation of bioanalytical results

**Fig. 1:**
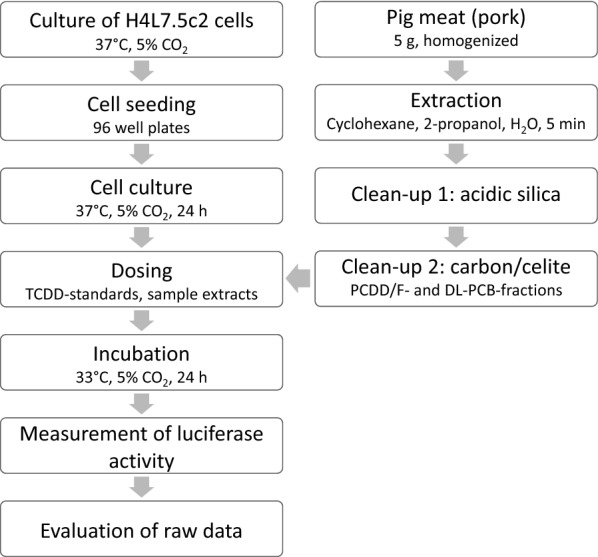
Outline of sample processing and measurement of luciferase activity using the CALUX bioassay

An outline of the CALUX bioassay procedure for sample analysis is presented in Fig. 1. H4L7.5c2 rat cells of defined sub-confluent density (60–70%) were harvested with trypsin and homogeneously suspended in cell culture medium (α-MEM) containing 10% (v/v) FBS. Cells were counted in a haemocytometer, then seeded in 96-well white clear bottom culture plates (Corning, New York, USA) at approximately 25,000 cells/well. After 1 h of pre-incubation at ambient conditions in a biosafety cabinet, the cells were grown for 20–24 h at 37 °C in the presence of 5% CO_2_.

Individual concentrations of a 2,3,7,8-TCDD-standard dilution series (generating assay concentrations of 0.0, 0.1, 0.3, 1.0, 3.0, 10, 30, 300, and 3000 pmol/L) and the sample extracts (both in DMSO) were first added to incubation medium and mixed, and the mixtures subsequently added in triplicate to 96-well plates containing H4L7.5c2 cells. The ability of the bioassay target compounds to adhere to the walls and bottom of the wells can lead to cells in individual wells of a triplicate being dosed with slightly different amounts of AhR-active compounds, leading to increased triplicate RSD values. Well-to-well variability of the cell response also further depends on edge effects commonly observed across 96-well microplates [42]. For a given cell system, inhomogeneous dosing of triplicates and edge effects are some of the main drivers for the assay working range, within which triplicate RSD values are subject to defined limitations (RSD < 15%). Especially, its bottom end should be as low as possible for the present application. Each batch of plates was therefore pre-tested for satisfactorily low and reproducible edge effects. The latter was further reduced by a 1 h pre-incubation under ambient conditions in the safety cabinet immediately after seeding the cells, resulting in an even distribution of the cells in each well. The plates were then placed in an incubator on a 1.5 mm-thin copper sheet on top of the incubator shelf. Copper not only has bactericidal properties, but being a fast heat conductor, it also helps to avoid the formation of patterns in the measured cell response (another type of "edge effects"), caused by temperature gradients near heat-radiating walls or by occasional opening of the incubator door. For preparing the dose media, we applied a validated, fast, and reproducible pipetting procedure followed by thorough re-mixing immediately before dosing the cells only into the inner 60 wells of 96-well plates. Cell culture medium was added to the cells in the 36 outer wells to maintain a balanced atmosphere above all wells allowing homogeneous evaporation rates across the plate during incubation. These and other precautions contributed greatly to triplicate RSDs generally being below 6% in various cell lines even at lower ends of assay working ranges.

Dosed cells were incubated at 33 °C in the presence of 5% CO_2_ for 24–48 h, depending on the time available for sample analysis. Previous studies have demonstrated a significant increase in luciferase activity when CALUX cells were incubated with test chemicals at 33 °C compared to 37 °C [43]. These cells are able to metabolically degrade a variety of AhR agonists (e.g., polycyclic aromatic hydrocarbons like benzo[a]pyrene) into inactive compounds. This feature is particularly effective during cell exposure, removing unstable AhR agonists, which despite all precautions may have made their way through the extraction and clean-up procedures into the final sample extract. Luciferase produced in response to these unstable agonists during the first hours of exposure is subsequently degraded by the cells during the 24 h exposure time that is generally observed before luciferase activity is measured [21]. In H4L7.5c2 cells, a prolonged incubation time of 48 h has shown to significantly reduce procedural blank values even further. The absence of cytotoxicity due to chemical treatment is confirmed by visual inspection of the cells prior to chemical addition and after the incubation. The cells were lysed, followed by addition of D-luciferin (Duchefa, Haarlem, The Netherlands), the substrate for the bioluminescence reaction of firefly luciferase. Luciferase activity was measured as emitted light (luminescence) from each well in a Centro LB 960 microplate luminometer (Berthold, Bad Wildbad, Germany) and activity expressed as relative light units (RLUs). Sample response data were converted to assay concentrations by comparison to a 2,3,7,8,-TCDD-standard curve. Results for each sample were then adjusted taking into account sample size and final extract volume, and subsequently corrected for the procedural blank and the apparent recovery of the positive control sample analyzed with each sample series [18].

To address the potential risk of a reduction in the cell response or even cytotoxicity due to co-eluted interfering compounds, which may lead to false-compliant results, but is likely to remain unnoticed, provisions were included in EU legislation [18]. Depending on laboratory experience, a certain percentage (2–10%) of samples declared compliant from screening shall be confirmed by a confirmatory method (e.g., GC/HRMS). A certain percentage of sample extracts (20%) shall further be measured in routine screening without and with 2,3,7,8,-TCDD added and the result evaluated for reduced response in comparison to the spiked amount.

### Concentration–response curves

A sound calibration curve is a prerequisite for sound assay performance. Special attention should therefore be paid to the quality of the fit, especially in the designated range of reportable results, to minimize lack-of fit errors. A key metric for evaluating suitability of the curve is the extent of agreement of nominal calibrator concentrations with back-calculated (back-fitted) concentrations read from the fitted curve. Differences between predicted and actual concentrations can be expressed as bias at each concentration level. This calibrator bias may either be calculated by averaging replicate RLU values before conversion (“RLU-based” bias), or by converting individual replicate RLUs to concentrations which are then averaged (“concentration-based” bias). Variability in the response data measured for each calibrator can be assessed as “RLU-based” RSD values [18]. Although replicate response data from the same 2,3,7,8-TCDD calibrator are statistically dependent, calibrator imprecision (generally termed “precision”) can also be expressed as “concentration-based” RSDs, as optionally suggested by Commission Regulation (EU) 2017/644 [18]: individual replicate RLUs are first converted to individual concentrations from which the RSD is calculated. Even though bias and imprecision associated with the calibrators will underestimate the true bias and imprecision when analyzing samples, they are a good upfront check of whether given requirements are met [41].

CALUX bioassay concentration–response data frequently follow the shape of a hyperbolic receptor-binding curve. This is despite the fact that the intracellular mechanisms involved in ligand-binding, ligand-dependent activation, and nuclear translocation of the liganded AhR, heterodimerisation of the ligand:AhR with the aryl hydrocarbon receptor nuclear translocator (ARNT) protein, binding of the resulting Ligand:AhR:ARNT complex to DRE-containing DNA, and induction of luciferase gene expression are complex inter-related processes. However, if the Hill coefficient (see below) is ≤ 1, then the curve will present an inflection point. When plotted on a semi-log scale to accommodate a full range of concentrations (i.e., from no response to maximal induction response), response data (relative light units, RLU) show a sigmoidal relationship to concentration.

#### The logistic model

**Fig. 2:**
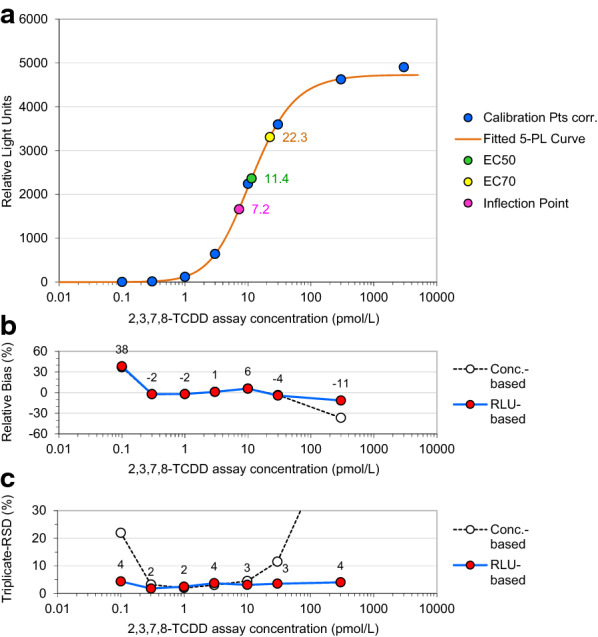
**a** 2,3,7,8-TCDD concentration–response curve obtained from H4L7.5c2 rat hepatoma cells with a 5-parameter logistic (5-PL) equation fitted to concentration–response data pairs by weighted sum of squared residuals regression (WSSR). Calibration Pts corr.: calibration data corrected for the assay background response. Maximum response: 4730 RLUs, minimum response: 0 RLU, EC50: 11.4 pmol/L, EC70: 22.3 pmol/L, inflection point: 7.2 pmol/L, slope: 1.17, asymmetry factor: 1.51. **b** Calibrator bias: RLU-based bias ≤ 11% in range 0.3–300 pmol/L, conc.-based bias ≤ 6% in range 0.3–30 pmol/L. **c** Calibrator imprecision: RLU-based RSD ≤ 4% in range 0.1–300 pmol/L, conc.-based RSD ≤ 12% in range 0.3–30 pmol/L. RLU-based (solid blue line): based on relative light units, Conc.-based (dotted black line): based on back-fitted concentrations

The 4-parameter logistic (PL) function is commonly used to produce a satisfactory depiction of the relationship between response (expressed in relative light units, RLUs) and analyte concentration. Mathematically analogous to *Hill’s* equation [44], the logistic function is utilized to fit the response data to a sigmoidally shaped line [45]. It defines a minimum response (*a*), the maximum response (*d*), the concentration required to evoke a response half-way between the minimum and maximum (*c*, being EC50), and a parameter that describes the steepness of the curve (*b*, Hill coefficient or Hill’s slope). However, we prefer a 5-PL function providing additional flexibility and the best possible quality-of-fit [46–48], especially in the lower end of the assay working range to the response data obtained in particular from H4L7.5c2 rat hepatoma cells, but from various other recombinant rat or mouse hepatoma cell lines, as well. Fitting 5-PL model functions to concentration–response data from our bioassays optimized for intra-assay (calibrator) precision resulted in assay working ranges expanded towards lower concentrations. Based on small relative bias and precision values of both the response data and reportable concentrations as seen in the bias and precision profiles in Figs. 2b and c, the use of the 5-PL model contributed to an enhanced accuracy of results in the range of interest, over the use of 4PL functions. This observation may be due to some degree of asymmetry being characteristic in principle of all sigmoid concentration–response curve shapes in bioassays to varying extent [47]. Resulting errors can be more significant than random variation yet easily accommodated by the 5-PL model function.

By adding an asymmetry parameter (*e*), this logistic model can handle asymmetric concentration–response data [47, 49, 50], allowing for asymmetric curves where EC50 is no longer the same as the inflection point, placing the latter at the actual point of transition from one asymptote to the other while approaching each asymptote at a different rate (Fig. 2a). Again, the parameter *a* represents the minimum response, *d* the maximum response, *b* the Hill coefficient, but *c* now signifies the inflection point and no longer the EC50. The formulas for the 4-PL [45, 48] and 5-PL [47, 48] model functions are shown below:

$$\begin{array}{*{20}c} {y = d + \frac{a - d}{{1 + \left( \frac{x}{c} \right)^{b} }}} &\quad {y = d + \frac{a - d}{{\left( {1 + \left( \frac{x}{c} \right)^{b} } \right)^{e} }}} \\\\ {4{ - }PL \, model \, function} &\quad {5{ - }PL \, model \, function} \\ \end{array} $$

#### Fitting the 5-PL model to heteroscedastic data

The noise (standard deviation, or variance) of bioanalytical response data generally increases with the response (heteroscedastic data). As a consequence, “low-quality” data points exhibiting higher variation generally found in the upper parts of concentration–response curves influence the fit to the same extent as “high-quality” data points typically located in the lower part of the curves. The quality of the fit may, therefore, be improved if heteroscedasticity is taken into account by placing less “weight” on responses exhibiting higher variation (“low quality” data) and giving more importance to “high quality” data points in the lower branch of the curve. Weighted sum of squared residuals regression (WSSR) reflects that the variance of response data is a function of the magnitude of the response [41]. As weighing factor, the inverse variance *w*_*i*_ = 1/variance (*y*_*i*_) of replicate response data at each concentration *i* is commonly used, *y*_*i*_ being the observed standard response, $\hat{y}_{i}$ the response predicted by the curve model, while *n* is the total number of concentration levels [47, 48]:

$${\text{WSSR}} = \mathop \sum \limits_{i = 1}^{N} w_{i} \left[ {y_{i} - \hat{y}_{i} } \right]^{2} .$$

Accurate weighing together with an appropriate model function frequently increases the range of reportable assay concentrations. Some concepts suggest to determine the true variance function from pooled historical assay data [47]. Alternatively, response variances can be assessed from six or more replicates at each calibrator concentration. Since it is impractical during routine analysis of large numbers of samples to run enough replicates within each assay to assess the true variance of the response for each calibrator, we used triplicate response RSDs as rough estimates [*w*_*i*_ = 1/RSD (*y*_*i*_)]. This seems justified for two reasons: First, relevant EU legislation [18] requires triplicate response RSDs to assess variability in response data. Second, within the range of reportable concentrations of fitted 5-PL functions, we regularly observe relative calibrator bias values significantly smaller than the ± 20% benchmark required for ligand-binding assay calibration curves by various regulatory agencies (US Food and Drug Administration (FDA), European Medicines Agency (EMA), Japanese Ministry of Health, Labor and Welfare (MHLW), Brazilian Sanitary Surveillance Agency (ANVISA)) [52].

Model curve parameters were optimized in an iterative process to achieve minimal bias of the calibrator concentrations over the maximum usable calibration range. Calculations on response data sets generated from series of 2,3,7,8-TCDD-standard dilutions were performed in custom-tailored data spreadsheets using the “Solver” add-on provided by Microsoft Excel (2016). Standard errors (SE) on parameter estimates were calculated with Data2Dynamics, a MATLAB toolbox originally designed for fitting ordinary differential equations to data [53], by the Institute of Medical Biometry and Statistics, Freiburg Center for Data Analysis and Modelling, University of Freiburg, Germany).

Goodness-of-fit tests evaluate how well a proposed model is consistent with a particular set of data, for which various methods are available [51, 54]. To compare the fit of the 4-PL and 5-PL models to our concentration–response data, we performed an extra sum-of-squares (ESS) *F* test [55] which is based on null hypothesis significance testing. The null hypothesis is that the simpler 4-PL model is correct. The ESS *F* test compares the two nested models differing by just one parameter, the simpler one (4-PL) being a special case of the more complex 5-PL, fit with WSSR. *F* quantifies the ratio between the relative increase in weighted sum of squared residuals and the relative increase in degrees of freedom (DF) [56]:

$$F = \frac{{\left( {{\text{WSSR}}_{{\text{4-PL}}} - {\text{WSSR}}_{{\text{5-PL}}} } \right)/{\text{WSSR}}_{{\text{5-PL}}} }}{{\left( {{\text{DF}}_{{\text{4-PL}}} - {\text{DF}}_{{\text{5-PL}}} } \right)/{\text{DF}}_{{\text{5-PL}}} }}.$$

It follows that if the 4-PL model is correct, *F* will be close to 1. On the other hand, if *F* is distinctly larger than 1, then the 5-PL model is correct; yet there is a chance that the 4-PL model is correct, but due to random variation the 5-PL model fit better. The *p* value indicates the probability for the latter scenario: a low *p* value suggests that the 5-PL fits better than the 4-PL. Results shown in Table 1 indicate that if the 4-PL model (the null hypothesis) were true, there would be a 1.42% chance of obtaining results that fit the 5-PL model (the alternative hypothesis) so well. Since the *p* value (0.0142) is less than the traditional significance level of 5%, we can conclude that the 5-PL model fit better to the concentration–response data obtained from H4L7.5c2 rat hepatoma cells than to the 4-PL model [56].

**Table 1:** Comparison of 4-PL and 5-PL models fit to concentration–response data obtained from H4L7.5c2 rat hepatoma cells with the extra sum-of-squares (ESS) *F* test

	Number of calibrators	Number of parameters	WSSR	DF
4-PL model (null hypothesis)	9	4	20.867	5
5-PL model (alternative hypothesis)	9	5	3.926	4
Difference			16.941	1
Difference (%)			431.51	25.00
Ratio (*F*)			17.26	
*p* value			0.0142	

It should be acknowledged that fitting algorithms may sometimes fail to result in the best 5-PL fit for two main reasons. Without a good choice of the starting points for coefficients *a* through *g*, in search for the nearest minimum, the algorithm may end up in one of multiple local minima in the fitting function. Second, strong coupling between coefficients of the 5-PL function may lead to local minima or into regions where the fitting curve is almost flat; some algorithms may stop there and again miss the global minimum. To our experience, this phenomenon may be overcome by covering the full range of the curve with a sufficient number of dilutions [47]. Observing these precautions, we successfully fitted many hundreds of asymmetric 5-PL model functions.

## Results and discussion

### Assessing concentration–response data

#### Calibrator bias and imprecision

**Fig. 3:**
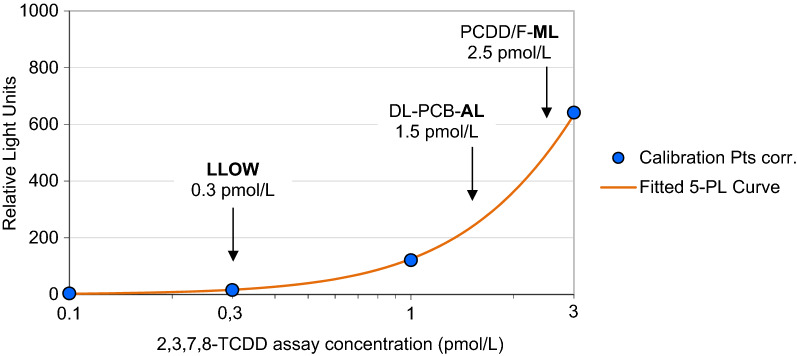
2,3,7,8-TCDD concentration–response curve obtained from H4L7.5c2 rat hepatoma cells, enlarged segment (0.1–3 pmol/L) from Fig. 2a. Lower limit of working range (LLOW, 0.3 pmol/L), and sample concentrations representing DL-PCB-AL (0.5 pg WHO-PCB-TEQ/g fat) and PCDD/F-ML (1.0 pg WHO-PCDD/F-TEQ/g fat), corresponding to assay concentrations of 1.5 pmol/L for DL-PCBs and 2.5 pmol/L for PCDD/Fs, respectively

WSSR regression aligns the calibration curve more closely to data of low variation (Figs. 2 and 3). The resulting improved fit exhibiting smaller calibrator bias values in particular in the lower part of the curve considerably reduces the risk for over- or underestimation of BEQ results in unknown food samples, especially in those with low MLs or ALs set by EU legislation. Figure 2a shows a 2,3,7,8-TCDD concentration–response curve obtained using highly sensitive “3rd generation” H4L7.5c2 rat hepatoma cells. Within the range of 0.3–300 pmol/L extending across three orders of magnitude, the relative RLU-based bias, for which no legal requirements have been established so far, does not exceed a ± 15% tolerance margin, while the more difficult to control concentration-based bias meets this criterion only in the smaller range of 0.3–30 pmol/L (Fig. 2b). To minimize the risk of false-positive and, more importantly in terms of consumer protection, false-negative results, and based on the experience with hundreds of bioassays performed on official food control samples, the authors recommend a 15% restriction also for the acceptable calibrator bias.

The EU legal requirement for tolerable imprecision of response data within the assay working range (RLU-based RSD < 15%) [18, 19] is met within a concentration range of 0.1–300 pmol/L, extending beyond three orders of magnitude (Fig. 2c, Table 2). Imprecision of back-fitted calibrator concentrations (concentration-based RSD) is more difficult to control and below 15% within the smaller range of 0.3–30 pmol/L. Compared to RLU-based RSDs, these concentration-based RSDs often tend to explode at the lower and upper ends of dose–response curves leading to smaller assay working ranges (Fig. 2c).

**Table 2:** Exemplary assay working ranges for 2,3,7,8-TCDD (in pmol/L) matching QC criteria for calibrator imprecision and bias derived from various CALUX cell lines after fitting 5-PL functions by WSSR regression

	H4L7.5c2 rat (20 DREs)	H4L1.1c4 rat (4 DREs)	DR-CALUX rat (4 DREs)	H1L7.5c3 mouse (20 DREs)	H1L6.1c2 mouse (4 DREs)
RLU-based RSD^a^ < 15%	0.1–300	0.3–300	0.3–300	0.1–300	0.3–300
Concentration-based RSD^b^ < 15%	0.3–30	1.0–30	0.3–30	0.3–10	1.0–100
RLU-based bias^c^ < 15%	0.3–300	0.3–30	0.3–30	0.3–30	1.0–100
Concentration-based bias^c^ < 15%	0.3–30	0.3–30	1.0–30	0.3–30	1.0–100
Assay working range A^d^	0.3–300	0.3–30	0.3–30	0.3–30	0.3–100
Assay working range B^e^	0.3–30	1.0–30	1.0–30	0.3–10	1.0–100
Source^f^	UCD	UCD	BDS	UCD	UCD

^a^Required according to EU legislation [18, 19]

^b^Recommended according to EU legislation [18, 19]

^c^Suggested by the authors

^d^Range A: fulfilling RLU-based criteria for precision and bias

^e^Range B: fulfilling RLU- and concentration-based criteria for precision and bias

^f^UCD: University of California Davis, Davis (USA); BDS: BioDetection Systems, Amsterdam (The Netherlands)

Sample concentrations equalling the action level set for DL-PCBs (0.5 pg WHO-PCB-TEQ/g fat), and the maximum level set for PCDD/Fs (1.0 pg WHO-PCDD/F-TEQ/g fat), correspond to assay concentrations of approximately 1.5 pmol/L (based on ~ 40% apparent recovery) for DL-PCBs, and of 2.5 pmol/L (based on ~ 80% apparent recovery) for PCDD/Fs, respectively (Fig. 3).

We compared both key indicators of assay performance, calibrator bias and imprecision, and the resulting working range for H4L7.5c2 rat hepatoma cells (this study) with those obtained using various other cell lines (4 DREs: H4L1.1c4 rat, DR-CALUX rat, H1L6.1c2 mouse hepatoma cells; 20 DREs: H1L7.5c3 mouse hepatoma cells) previously evaluated [41] (Table 2). Commercially available DR-CALUX cells, a recombinant rat hepatoma (H4IIe)-based cell line (H4L1.1c10), were obtained from BioDetection Systems (The Netherlands). When using CALUX cell lines that contain 4 DREs, the lower limit of working range (LLOW), or the concentration above which results may be reported [18], is typically close to 1 pmol/L if both RLU-based and concentration-based criteria for bias and imprecision are fulfilled (assay working range B in Table 2) [23, 41]. Calibrator bias and imprecision obtained in this study using H4L7.5c2 cells that contain 20 DREs, however, reveal that the LLOW shifted to significantly smaller assay concentrations as low as 0.3 pmol/L with a working range extending across two orders of magnitude (Figs. 2 and 3, Table 2). When utilizing H1L7.5c3 mouse hepatoma cells, which also contain 20 DREs, the LLOW equals 0.3 pmol/L, as well. However, the range in which assay concentrations match all criteria for bias and imprecision is smaller with an upper limit of working range (ULOW) of just 10 pmol/L. It follows that the “3rd generation” H4L7.5c2 rat hepatoma cells provide the most suitable working range of the assay for low sample concentrations, regardless of whether only the current EU RLU-based imprecision requirements are met or whether bias-related and concentration-based criteria are additionally observed (Table 2). Our results, which are exemplary for hundreds of assays performed with the five different cell lines evaluated in this study, demonstrate that the blanket use of the EC70 value (Table 3) as ULOW, as required by current EU legislation [18, 19], unnecessarily but significantly narrows the upper parts of the working ranges.

**Table 3:** Key parameters of 5-PL functions fitted by WSSR regression to fold induction/concentration data pairs obtained from various CALUX cell lines incubated with increasing concentrations of 2,3,7,8-TCDD

Parameters	H4L7.5c2 rat (20 DREs)	H4L1.1c4 rat (4 DREs)	DR-CALUX rat (4 DREs)	H1L7.5c3 mouse (20 DREs)	H1L6.1c2 mouse (4 DREs)	Unit
Number of calibrators	9	8	8	9	9	
Number of parameters	5	5	5	5	5	
Degrees of freedom (DF)	4	3	3	4	4	
WSSR	3.9258	1.4241	0.2661	0.5751	3.8570	
Chi-square-to-DF ratio, *χ*^2^/DF	0.9814	0.4747	0.0887	0.1438	0.9642	
Maximum fold induction	136	20.6	8.75	10.5	31.2	
SE of max. fold induction	3.26	1.14	0.29	0.40	0.63	
EC70	22.3	19.8	14.0	9.21	50.5	pmol/L
EC50	11.4	5.52	7.89	5.28	23.5	pmol/L
Inflection point (*c*)	7.22	3.67	12.0	4.63	15.0	pmol/L
SE of *c*	0.74	2.49	1.60	1.20	3.57	pmol/L
Slope (*b*)	1.17	0.90	1.70	1.48	1.03	
SE of *b*	0.08	0.17	0.28	0.22	0.09	
Asymmetry factor (*e*)	1.51	1.81	0.63	1.15	1.42	
SE of *e*	0.15	0.96	0.14	0.32	0.26	
Fold induction at 1.0 pmol/L	4.47	2.44	1.53	1.62	1.56	
Fold induction at 0.3 pmol/L	1.46	1.33	1.16	1.10	1.12	
Background contribution 50%^a^	0.46	0.74	1.78	1.37	1.60	pmol/L
Minimum fold induction	1.00	1.00	1.00	1.00	1.00	

^a^Concentration at which the relative unspecific background contribution to results equals 50% (see Fig. 4c)

### Fold induction and unspecific assay background contribution to results

While absolute induction is driven by the efficiency of gene expression, fold induction is the ratio between the cell response (RLU) induced by a calibration standard, or by AhR-active dioxin-like compounds present in a sample extract, and the unspecific assay background response (RLU). Table 3 shows key parameters of 5-PL functions fitted by WSSR regression to fold induction/concentration data pairs obtained from various recombinant CALUX cell lines. Furthermore, the Chi-square-to-degrees of freedom ratio (*χ*^2^/DF, with *χ*^2^ ~ WSSR) known to be a stand-alone index characterizing the quality of a model fit is provided for each cell line [57]. Some scientists suggest a benchmark value of 2 for a good fit, while 3 indicates an acceptable value [58]; while others explain that this ratio should be close to or below 1, if the standard deviations used for fitting are a good representation of the disagreement between data and fit [59, 60]. The latter is the case for each cell line (Table 3). In some cases, the *χ*^2^/DF value is considerably smaller than 1 which suggests an overestimation of the data’s standard deviation [60]. An explanation can be found in the fact that triplicate standard deviations were used as rough estimates.

**Fig. 4:**
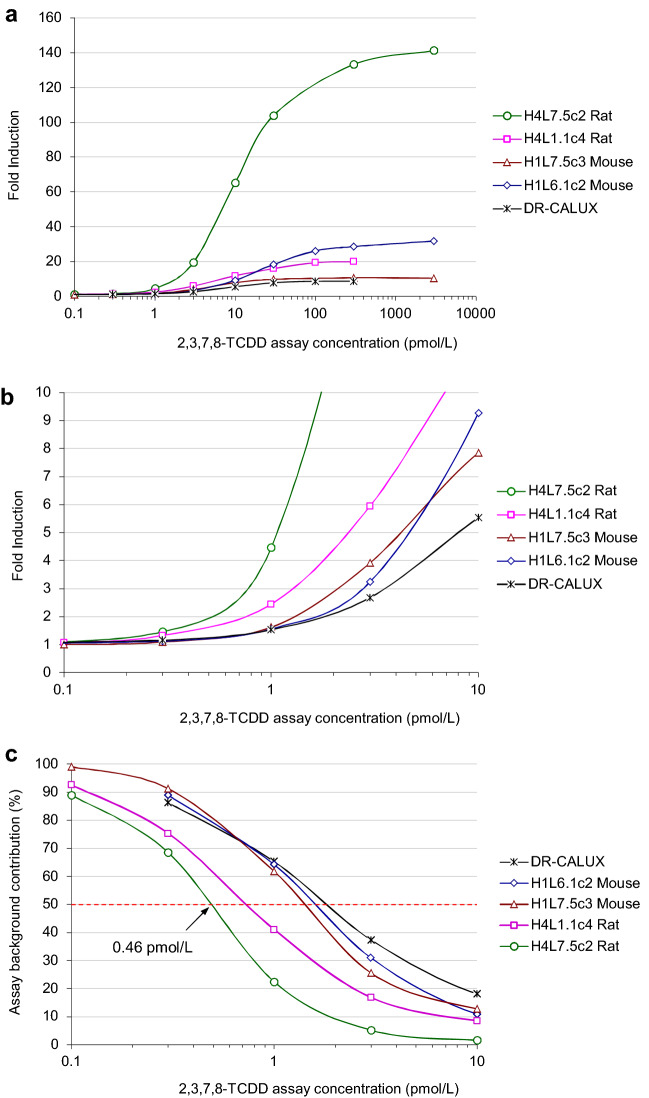
2,3,7,8-TCDD concentration–response analysis with recombinant CALUX cell lines. **a** Fold induction vs assay concentrations for the indicated CALUX hepatoma cell lines. **b** Enlarged segment (range: 0.1–10 pmol/L). **c** Relative unspecific assay background contribution at low assay concentrations for various recombinant CALUX cell lines (range: 0.1–10 pmol/L) (see Table 4). The dotted red line indicates 50% unspecific assay background contribution to results

The resulting curves are depicted in Fig. 4a and b. EC70, EC50, inflection point (c), slope (b), and asymmetry factor (e) values are identical to those calculated from the respective concentration–response curves. While the minimum fold induction equals 1 by definition, maximum fold induction differs considerably between the various cell lines and so does the fold induction at low assay concentrations. Table 3 shows that among all cell lines compared, highest fold induction values exemplary calculated at 0.3 and 1.0 pmol/L were 1.46 and 4.47, respectively, obtained using the H4L7.5c2 rat hepatoma cells.

The relative unspecific assay background contribution to bioanalytical results inversely reflected in the respective fold induction should be well controlled especially in the lower part of the assay working range. For the CALUX cell lines compared, Table 4 shows the relative unspecific assay background contribution at various calibrator concentrations; corresponding curves are depicted in Fig. 4c. While at 1 pmol/L, for example, the relative background contribution may be as high as 65% of the response, it is only 22% for H4L7.5c2 rat hepatoma cells. However, at an assay concentration of 0.3 pmol/L, only about 10% of the result is based on a response to AhR-active dioxin-like target compounds in some cell lines, while the remaining 90% must be attributed to unspecific background response. Again, the H4L7.5c2 rat hepatoma cells show the best performance, with 69% unspecific response and 31% of the response based on AhR-active dioxin-like target compounds.

**Table 4:** Relative unspecific assay background contribution to bioanalytical results (in %) at calibrator concentrations in various CALUX cell lines (see Fig. 4c)

TCDD (pmol/L)	H4L7.5c2 rat (20 DREs)	H4L1.1c4 rat (4 DREs)	DR-CALUX rat (4 DREs)	H1L7.5c3 mouse (20 DREs)	H1L6.1c2 mouse (4 DREs)
0.1	89	93	–	99	–
0.3	69	75	86	91	89
1.0	22	41	65	62	64
3.0	5.2	17	37	26	31
10	1.5	8.5	18	13	11
30	1.0	6.3	13	10	5.5
100	–	5.2	11		3.8
300	0.8	5.0	11	9.4	3.5
3000	0.7	–	–	9.6	3.1

These examples show that a restriction should be introduced to the unspecific background response to ensure appropriate background correction to the cell response measured for calibrator standards and sample extracts of each assay. We, therefore, propose that, in addition to restrictions to calibrator bias and imprecision, the relative background contribution to results should be limited to 50% within assay working ranges. Based on this requirement, LLOWs achieved with recombinant CALUX hepatoma cell lines investigated in this study are between 0.46 pmol/L for the H4L7.5c2 rat cells and 1.77 pmol/L for DR-CALUX rat cells (Table 3, Fig. 4c).

### Method validation

Basic method performance, in particular efficiency and reproducibility of the extraction, purification, and assay procedures, was evaluated during an initial validation by matrix-matched calibration experiments [22]. Thirty identical, homogenized confirmed “blank” pig meat samples (0.093 pg WHO-PCDD/F-TEQ/g fat, 0.068 pg WHO-PCB-TEQ/g fat, 0.160 pg WHO-PCDD/F-PCB-TEQ/g fat) were each spiked with 2,3,7,8-TCDD around the ML set for PCDD/Fs (PCDD/F-ML; 1.0 pg WHO-PCDD/F-TEQ/g fat), and with PCB 126 around the AL set for DL-PCBs (DL-PCB-AL; 0.5 pg WHO-PCB-TEQ/g fat). Six series of 5 samples each were thus prepared with concentrations at around 0x, 0.5x, 1x, 1.5 ×, and 2 × ML, or AL (see “calibrated range” in Table 5). Spiking of pre-analyzed “blank” samples instead of using incurred materials ensures that the cell response is not significantly influenced by co-extracted non-regulated AhR agonists, but is due to AhR-active dioxin-like compounds present in the sample extract. Samples were submitted to extraction, clean-up, and measurement of luciferase activity as described above in six consecutive series under within-laboratory reproducibility conditions for the six repetitions on each level [22]. Within-lab reproducibility (intermediate precision) expressed as RSD_Rw_ is the precision obtained within a single laboratory over a longer time period, taking into account more changes than repeatability. Sample concentrations (in BEQs) were calculated by comparison of the cell response with a 2,3,7,8-TCDD calibration curve and correction of the result for the blank BEQ level and the “apparent recovery” of a reference sample spiked at ML or AL [18, 22].

**Table 5:** Initial validation: linear regression on BEQ/TEQ results from bioanalytical screening of 30 “blank” pig meat (pork) samples spiked around MLs and ALs, for PCDD/Fs and DL-PCBs followed by calculation of the respective cut-off concentrations [18, 22]

Parameter	Unit	Symbol	PCDD/Fs (ML)	PCDD/Fs (AL)	DL-PCBs	PCDD/Fs + DL-PCBs
Number of calibration levels		c	5	5	5	5
Number of replicates per level		m	6	6	6	6
Number of calibration points		n	30	30	30	30
Calibrated range	pg TEQ/g fat	R	0.09–1.99	0.09–1.99	0.07–1.03	0.16–3.02
Slope, calibration line		b	0.913	0.913	0.578	0.795
Standard error of the slope		s_b_	0.065	0.065	0.044	0.048
Y-intercept, calibration line	pg BEQ/g fat	a	0.022	0.022	0.180	0.212
Standard error of the y-intercept	pg BEQ/g fat	s_a_	0.077	0.077	0.027	0.086
Coefficient of correlation		r	0.9354	0.9354	0.9280	0.9518
Critical value for r (df = *n* *−* 2, *p* = 0.05, two-sided)		crit_*r*_	0.3610	0.3610	0.3610	0.3610
Square coefficient of correlation		r^2^	0.8750	0.8750	0.8612	0.9060
GC/HRMS decision limit^a^	pg TEQ/g fat	DL	1.2	0.90	0.60	1.50
BEQ concentration at DL	pg BEQ/g fat	y_DL_	1.1	0.84	0.53	1.40
Mean recovery^b^ at ML, or AL		%	84.2	82.2	92.1	86.9
Residual standard deviation^c^	pg BEQ/g fat	*s*_*yx*_	0.2261	0.2261	0.0774	0.3186
Relative *s*_*yx*_ at *y*_DL_ (precision^c^)	%	*s*_*yx,rel*_	20.3	26.8	14.7	18.0
Cut-off concentration	pg BEQ/g fat	y_Cut-off_	0.83	0.56	0.43	1.1
Maximum level	pg TEQ/g fat	ML	1.0	–	–	1.25
Action level	pg TEQ/g fat	AL	–	0.75	0.50	–

^a^ML, or AL, plus the expanded measurement uncertainty U (here: U = 20%)

^b^After correction for the blank BEQ level and the apparent recovery of a reference sample spiked at ML, or AL

^c^Within-laboratory reproducibility conditions

**Fig. 5:**
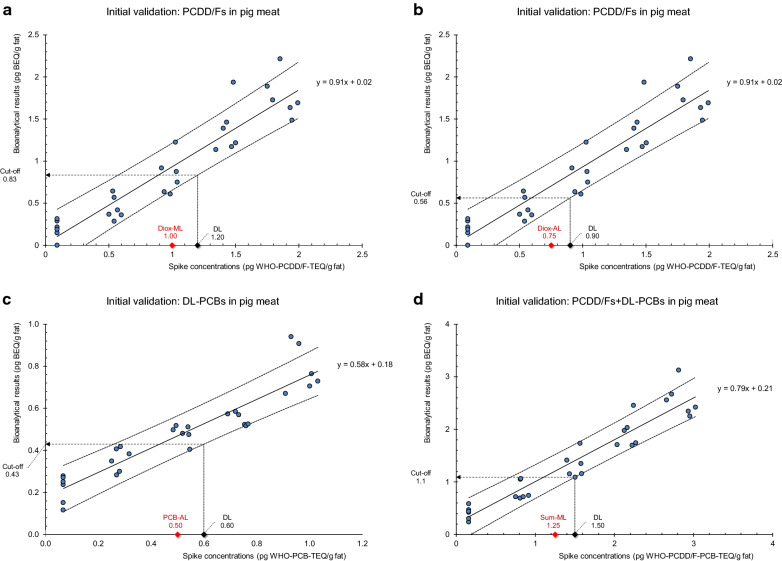
Initial validation: BEQ/TEQ calibration data from bioanalytical screening of 30 “blank” pig meat samples spiked around MLs and ALs. Regression lines, 99% prediction intervals, MLs, ALs, DLs (based on U = 20%), and derived cut-off concentrations are indicated. **a** PCDD/Fs, ML, **b** PCDD/Fs, AL, **c** DL-PCBs, AL, and **d** sum of PCDD/Fs and DL-PCBs, ML. “Spike concentrations” means GC/HRMS results of the blank samples plus the respective calculated spiking concentrations (TEQ). Linear regression results are presented in Table 5

For PCDD/Fs, DL-PCBs, and the calculated sum of PCDD/Fs and DL-PCBs, bioanalytical results (BEQ) were plotted vs the spiking levels (TEQ), equalling the GC/HRMS results of the blank samples plus the respective calculated spiking concentrations (TEQ) (Fig. 5a–d). In GC/HRMS confirmatory analysis, only those sample results exceeding the ML (or AL) plus the expanded measurement uncertainty U are considered noncompliant. Bioassay cut-off concentrations are therefore based on the GC/HRMS decision limit (DL = ML + U). Cut-off values were calculated from the lower band of the prediction interval at DL as the BEQ level above which 99% (required: 95% [18]) of the area of the assumed normal distribution curve of the response variables corresponding to DL is located [22].

Correlation coefficients (r = 0.93–0.95) indicate a high positive linear relationship between BEQ and TEQ results within each target analyte group (PCDD/Fs, DL-PCBs, and the calculated sum of PCDD/Fs and DL-PCBs) (Table 5). Calibration line slopes, being measures of method sensitivity, are 0.91 for PCDD/Fs, 0.58 for DL-PCBs, and 0.79 for the sum of PCDD/Fs and DL-PCBs. The reduced slope in DL-PCBs may be due to the CALUX system’s low relative response to PCB 126 (approximately 40%) compared to its response to 2,3,7,8-TCDD. As expected, *y*-intercepts representing mean “blank” sample concentrations (in BEQ) are close to the unspiked sample concentrations (in TEQ).

Commission Regulation (EU) 2017/644 lays down acceptable ranges for “apparent recoveries” in QC samples calculated at MLs and ALs, expressed as percentage of the BEQ level in comparison to the TEQ level: for DL-PCBs 20–60%, for PCDD/Fs 50–130%, and for the sum of PCDD/Fs and DL-PCBs 30–130% [18]. Mean recoveries at MLs and ALs being estimates of systematic errors are in a satisfactory range of 82–92% (Table 5). The higher recovery found for DL-PCBs when compared to the legal requirement is due to the elevated mean matrix blank of 0.18 pg BEQ/g fat. Within-laboratory reproducibility, for which the restriction is 30% [18], was calculated as relative residual standard deviation of BEQ results at the respective GC/HRMS decision limits (DLs) over the exceedance of MLs and ALs (DL_ML_ = ML + U; DL_AL_ = AL + U) [22]. For PCDD/Fs, this precision parameter turned out to be 20.3% at the PCDD/F-DL_ML_ and 26.8% at the PCDD/F-DL_AL_, for DL-PCBs 14.7% at the DL-PCB-DL_AL_, and for the sum of PCDD/Fs and DL-PCBs 18.0% at the PCDD/F-PCB-DL_ML_. Cut-off concentrations above which samples are declared “suspected to be noncompliant” (Table 5) are 83% of the PCDD/F-ML, 88% of the PCDD/F-PCB-ML, 75% of the PCDD/F–AL and 86% of the DL-PCB-AL, and thus reasonably close to the respective legal limit values.

### Performance evaluation with authentic samples

**Fig. 6:**
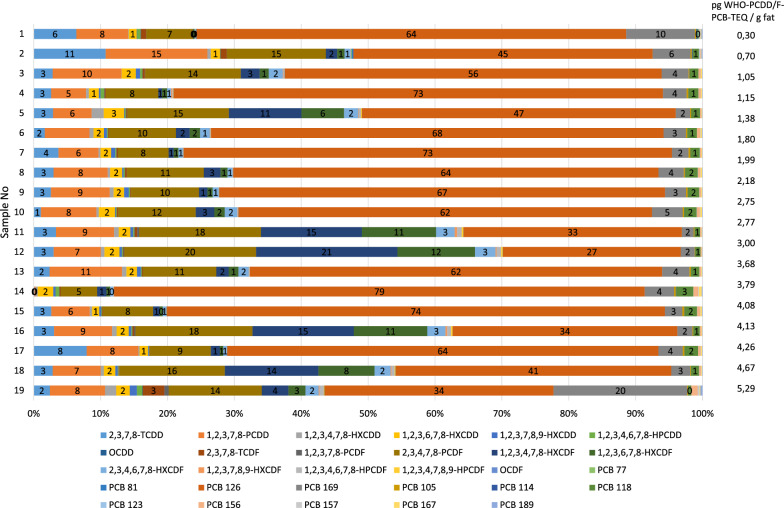
Relative contribution (in %) of 29 individual PCDD/F and DL-PCB congeners to WHO-PCDD/F-PCB-TEQ in 19 pig meat samples

Meat samples collected from official routine control and from contamination incidents involving PCDD/Fs and DL-PCBs were screened with the CALUX bioassay as described above. Based on their degree of contamination results from 19 of these samples, six of which were screened in duplicate (*n* = 25 total screening results) were selected to be included in the re-evaluation of method performance under “real-life” conditions [18, 22]. GC/HRMS analysis had revealed that these samples, representing a variety of congener patterns (Fig. 6) and physico-chemical properties (e.g., extractable lipids: 8.2–38.2%), were contaminated with PCDD/Fs and DL-PCBs in a range from the respective limits of quantification (LOQs) of the confirmatory method to almost three times the PCDD/F-ML, almost six times the AL set for DL-PCBs and about four times the PCDD/F-PCB-ML (see “calibrated range” in Table 6). Results were collected in a BEQ/TEQ database for quality control purposes as required by EU legislation [18, 22].

**Table 6:** Performance re-evaluation: linear regression on results from bioanalytical screening (BEQ) of 25 pig meat (pork) samples for PCDD/Fs and DL-PCBs, and from GC/HRMS analysis (TEQ), followed by calculation of the respective cut-off concentrations, and of α- and ß-errors (false-noncompliant and false-compliant rates) [18, 22]

Parameter	Unit	Symbol	PCDD/Fs (ML)	PCDD/Fs (AL)	DL-PCBs	PCDD/Fs + DL-PCBs
Number of samples		N	19	19	19	19
Number of analyses per sample^a^		m	1 (6 × 2)	1 (6 × 2)	1 (6 × 2)	1 (6 × 2)
Number of analyses (total)		n	25	25	25	25
Calibrated range	pg TEQ/g fat	R	0.07–2.86	0.07–2.86	0.23–3.59	0.30–5.67
Slope, calibration line		b	0.904	0.904	0.722	0.891
Standard error of the slope		s_b_	0.050	0.050	0.032	0.029
*y*-intercept, calibration line	pg BEQ/g fat	a	0.092	0.092	0.134	− 0.060
Standard error of the *y*-intercept	pg BEQ/g fat	s_a_	0.075	0.075	0.063	0.093
Coefficient of correlation		r	0.9665	0.9665	0.9784	0.9884
Critical value for r (df = *n* − 2, *p* = 0.05, two-sided)		crit_*r*_	0.3961	0.3961	0.3961	0.3961
Square coefficient of correlation		r^2^	0.9342	0.9342	0.9573	0.9770
GC/HRMS decision limit^b^	pg TEQ/g fat	DL	1.2	0.90	0.60	1.50
BEQ concentration at DL	pg BEQ/g fat	y_DL_	1.2	0.91	0.57	1.28
Residual standard deviation^c^	pg BEQ/g fat	*s*_*yx*_	0.2326	0.2326	0.1504	0.2155
Relative *s*_*yx*_ at *y*_DL_ (precision^c^)	%	*s*_*yx,rel*_	19.8	25.7	26.5	16.9
Cut-off concentration	pg BEQ/g fat	y_Cut-off_	0.70	0.42	0.24	0.82
α-Error (false-noncompliant rate)^d,e^	%	α	17 / 0	38 / 24	0 / 0	10 / 8
ß-Error (false-compliant rate)^d^	%	ß	0	0	0	0
Maximum level	pg TEQ/g fat	ML	1.0	–	–	1.25
Action level	pg TEQ/g fat	AL	–	0.75	0.50	–

^a^Six out of 19 samples were analysed in duplicate

^b^ML, or AL, plus the expanded measurement uncertainty U (here: U = 20%)

^c^Within-laboratory reproducibility conditions

^d^From application of the cut-off value established during initial validation

^e^Based on results potentially noncompliant from screening/based on all screening results

**Fig. 7:**
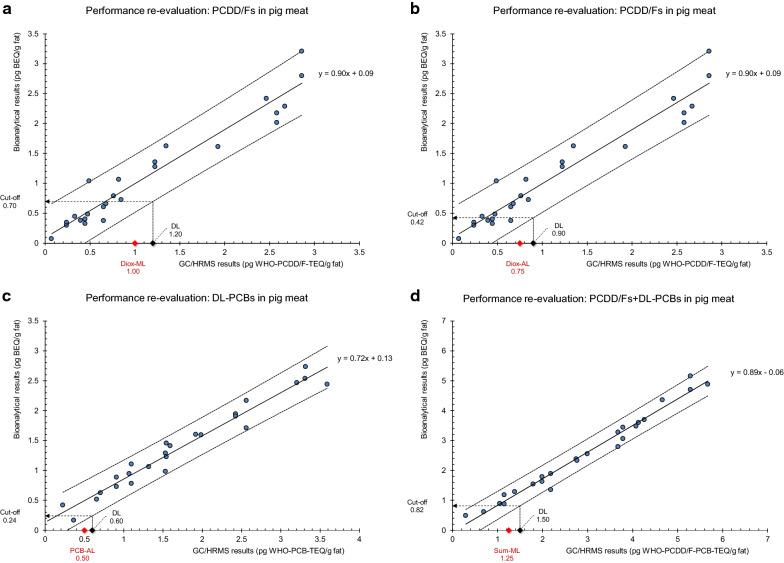
Performance re-evaluation: BEQ/TEQ calibration data from bioanalytical screening and GC/HRMS analysis of 25 contaminated pig meat samples. Regression lines, 99% prediction intervals, MLs, ALs, DLs (based on U = 20%), and derived cut-off concentrations are indicated. **a** PCDD/Fs, ML, **b** PCDD/Fs, AL, **c** DL-PCBs, AL, and **d** sum of PCDD/Fs and DL-PCBs, ML. Linear regression results are presented in Table 6

Bioanalytical results (BEQs) were plotted vs the corresponding GC/HRMS-TEQs for PCDD/Fs, DL-PCBs, and for the calculated sum of PCDD/Fs and DL-PCBs, to re-calibrate the bioassay with authentic incurred sample material. Regression lines and 99%-prediction intervals were calculated from which various key performance parameters derived such as intermediate precision and recoveries for the target analyte groups, cut-off concentrations, and possible false-noncompliant (α-error) and false-compliant rates (ß-error) resulting from application of the initially calculated cut-offs during routine screening (Fig. 7a–d, Table 6).

Despite the variability in concentrations, congener patterns (Fig. 6) and physico-chemical properties such as extractable lipids, the BEQs, and corresponding TEQ values measured for PCDD/Fs, DL-PCBs, and PCDD/F-PCBs seem to be even more strongly related (*r *= 0.98, 0.98, 0.99, respectively) than in the spiked samples. The high degree of correlation suggests that PCDDs, PCDFs, and PCBs were mainly responsible for the induction response and any other AhR-active chemicals (e.g. brominated dibenzo-p-dioxins, furans and biphenyls, and other dioxin-like compounds) played a minimal role. However, this should also be the case within the initial spiking experiments involving just one “blank” sample. The slightly higher coefficients of correlation achieved for incurred samples may partly be due to significantly wider calibration ranges involved than usually established during initial spiking experiments [18].

In principle, comparison of a sample concentration–response curve to a 2,3,7,8-TCDD-standard curve can reveal whether the extracts of the meat samples differing considerably in congener patterns (Fig. 6) nevertheless behave as dilutions of 2,3,7,8-TCDD. If both curves are parallel, differing only by a horizontal shift, then it is likely that the compounds present in a sample extract that produce a response in the assay meet all requirements of the TEQ principle [18]. If the slope parameters are not equivalent, then BEQs estimated at various percentages of the maximal effective concentration (e.g., EC20, EC50, or EC70) would differ in the two curves. However, the highest PCDD/F- and DL-PCB concentrations of the samples included in this study equal assay concentrations of just 5.4 and 7.2 pmol/L, respectively. Since the highest calibrator concentration is 3000 pmol/L, concentration–response curves could not be established for these samples.

Calibration line slopes close to 0.90 for both PCDD/Fs and the sum of PCDD/Fs and DL-PCBs are well within the acceptable range [18] indicating a high degree of method sensitivity similar to that previously obtained from the spiking experiments. *y*-intercepts, or mean sample “blank” values, are close to zero or to the smallest sample concentrations, respectively (Table 6). This allows interpretation of the slope values as an approximate recovery of the individual analyte target groups (90% for PCDD/Fs and the sum of PCDD/Fs and DL-PCBs). As expected based on the initial validation, the slope for DL-PCBs is with 0.72 corresponding to about 70% recovery for DL-PCBs again smaller yet slightly beyond the expected recovery range [18], although not to the same extent as during spiking experiments, an observation which may be caused by other DL-PCBs present in the incurred samples’ extracts.

Within-laboratory reproducibility calculated as relative residual standard deviation of BEQ results at the respective GC/HRMS decision limits (DLs) is 19.8% for the PCDD/F-DL_ML_, 25.7% for the PCDD/F-DL_AL_, 26.5% for the DL-PCB-DL_AL_, and 16.9% for the PCDD/F-PCB-DL_ML_ (Table 6), indicating that method precision is fully compliant with legal requirements (RSD_R_ < 30%) [18, 22]. Measurement uncertainties (MU) were not calculated for reasons explained elsewhere [35].

Cut-off concentrations are based on an interplay of various parameters, mainly regression line slope and *y*-intercept, GC/HRMS decision limit (based on the variability in measured TEQ results), and the variability of bioanalytical results. In general, cut-offs are lower when established from incurred samples than those obtained from spiking experiments, which was also the case within this study: 70% (previously 83%) of the PCDD/F-ML, 66% (previously 88%) of the PCDD/F-PCB-ML, 56% (previously 75%) of the PCDD/F–AL, and 48% (previously 86%) of the DL-PCB-AL (Tables 5 and 6).

Consequently, initial cut-off concentrations require adjustment by substituting them with the values obtained from re-calibration, to keep the risk for false-compliant results as low as possible at all times. Moreover, the actual rates of false-noncompliant (α-error) and false-compliant results (ß-error) possibly generated by applying the initial cut-off value in routine screening require inspection. When checking compliance with MLs, a false-compliant rate of ß < 5% must always be ensured [18, 22], which is indeed the case for all target analyte groups included in this study. The rate of false-noncompliant results being the fraction of results suspected to be noncompliant from screening but after follow-up confirmatory analysis found to be compliant must not exceed an acceptable percentage. However, no legal restriction exists [18]. Within this study, the fraction of false-noncompliant results based on the number of suspected samples varies between 0% based on the DL-PCB-AL, and 38% based on the PCDD/F–AL (Table 6).

However, if the advantageousness of the bioassay shall be assessed, the fraction of false-noncompliant results shall always be compared with the total number of screened samples. This rate must be low enough to render the bioanalytical screening beneficial and economically worthwhile [18, 22]. The α-error when based on the 25 screened samples included in this study is 0% each for PCDD/Fs (ML) and DL-PCBs (AL), 8% for the sum of PCDD/Fs and DL-PCBs (ML), and 24% for PCDD/Fs (AL), which significantly reduces the workload of the downstream confirmatory analytics. Additional samples had been screened which were contaminated at higher levels rendering them not suitable to be included in this study; all sample concentrations were above the respective MLs, and/or ALs, so that the actual false-noncompliant rates based on all screened samples (*n* = 52) and target analyte groups were below 12%.

### Turn-around times and sample throughput

The method described here enables one laboratory technician to process a series of 12 samples, for example, starting from weighing via lipid extraction, purification of the extracts, and measurement of luciferase activity to statistical evaluation of the raw data within 52 h. If two series of 10–12 samples are processed each week, 840–1000 samples can be analyzed by one laboratory assistant per year (based on 42 working weeks). The advantage of such accelerated methods for dioxins and PCBs screening is economically obvious in routine analysis and during the so-called “contamination incidents”, when large numbers of samples must be analysed within very short time periods to enable detection of sources and paths of contamination as quickly as possible [20, 35]. After eliminating the source, monitoring of concentration levels for weeks or even months may eventually lead to official release of previously banned products when results fall below certain limits.

## Conclusions

Under the EU’s General Food Law’s legislative framework [61], member states are required to monitor a wide range of food samples for dioxins and DL-PCBs, some—such as pork—at low levels of contamination. In response, we developed a fast, efficient, and cost-effective bioanalytical routine method implemented upstream of the confirmatory GC/HRMS technology. Based on a rapid extraction step followed by selective clean-up, it uses a highly responsive “3rd generation” H4L7.5c2 recombinant rat hepatoma CALUX cell line. Our procedure reliably identifies pork samples and, in principle, any other EU-regulated foods of animal origin [35], with concentrations suspected to exceed the specified maximum and action levels. It can cope with small sample amounts (5–10 g) and allows an annual throughput of up to 1000 samples per lab assistant, at turn-around times of ~ 52 h for 10 and more samples per technician.

Well-controlled relative bias and precision values of response data and reportable concentrations together with a reduced unspecific background response resulted in a threefold shift of the lower end of the assay working range towards lower concentrations, if compared to CALUX cell lines containing only 4 DREs. We suggest including a 15% restriction for the calibrator bias in relevant EU legislation [18] to minimize the risk for false-compliant/noncompliant results. The contribution of the relative unspecific assay background response should also be limited, i.e., to 50% of the response measured for reportable concentrations. Obtained performance parameters demonstrate that the H4L7.5c2 rat hepatoma cell line used in this study is, to date, the most suitable CALUX cell bioassay applied for official food control.

Between the BEQ and TEQ results, we found a strong uphill linear relationship, respectively, for PCDD/Fs, DL-PCBs, and the sum of PCDD/Fs and DL-PCBs. The method was fully validated according to the EU’s legal requirements [18] and re-calibrated using authentic pork samples. False-compliant rates (ß-errors) of 0% each for PCDD/Fs, DL-PCBs, and the sum of PCDD/Fs and DL-PCBs together with false-noncompliant rates (α-errors) below 12% demonstrate the advantageousness [18] of the bioassay. High bioanalytical performance at low levels of contamination is of special significance against the background of the European Union strategy aimed at reducing levels of contaminants in the environment, feed, and food to ensure a high level of public health protection [20, 32], which may result in a further reduction of the current MLs and ALs in the future.

### Outlook

As a next step, the validated 96-well formatted assay should be adapted to a 384-well format. The enhanced AhR induction response in the H4L7.5c2 recombinant rat hepatoma cells makes them particularly useful for high-throughput screening purposes using 384-well plates, because significantly fewer cells are needed to obtain a measurable response [39]. In fact, a “3rd generation” AhR-responsive human CALUX cell line has been successfully utilized in a 1536-well plate format for high-throughput screening of a large chemical library for AhR agonists [62]. However, because the wells of a 384-well plate are clustered together, potential cross-contamination between adjacent wells must be thoroughly investigated, along with edge effects and well-to-well variability. Additionally, finding a suitable serum-free formulation may be very challenging. However, variability in composition, costs, and potential contamination with viruses or prions has more recently become a driving force for attempts to substitute bovine serum as an essential component in culture media with a more defined and animal-component free medium, thus creating an ethical environment for bioanalytical screening.

## Data Availability

The datasets used and/or analysed during the current study are available from the corresponding author on reasonable request.

## Abbreviations

AhR: Aryl hydrocarbon receptor
AL: Action level
ANVISA: Brazilian Sanitary Surveillance Agency
ARNT: Hydrocarbon receptor nuclear translocator
BEQ: Bioanalytical equivalent
BSS: Balanced salt solution
bw: Body weight
CALUX: Chemically Activated Luciferase gene eXpression
CONTAM: EFSA Panel on Contaminants in the Food Chain
ddH_2_O: Double-distilled water
DF: Degrees of freedom
DL: Decision limit
DNA: Deoxyribonucleic acid
DRE: Dioxin responsive element
EC50: Effective concentration 50, half maximal effective concentration
EC70: Effective concentration 70, 70% of maximal effective concentration
EDTA: Ethylenediaminetetraacetic acid
EFSA: European Food Safety Authority
EMA: European Medicines Agency
ESS: Extra sum of squares
EU-RL: European Union Reference Laboratory
FBS: Fetal bovine serum
FDA: US Food and Drug Administration
GC: Gas chromatography
GC/HRMS: Gas chromatography coupled to high-resolution mass spectrometry
Hsp90: Heat shock protein 90
HRMS: High-resolution mass spectrometry
LLOW: Lower limit of working range
LOQ: Limit of quantification
MEM: Minimum essential medium
MHLW: Japanese Ministry of Health, Labor and Welfare
ML: Maximum level
MU: Measurement uncertainty
p23: Prostaglandin E synthase 3 (PTGES3)
PBS: Phosphate buffered saline
PCB: Polychlorinated biphenyl
DL-PCB: Dioxin-like polychlorinated biphenyl
DL-PCB-AL: AL set by the EU for the sum of DL-PCBs
PCDD: Polychlorinated dibenzo-p-dioxin
PCDD/F-ML: ML set by the EU for the sum of PCDDs and PCDFs
PCDD/Fs: Polychlorinated dibenzo-p-dioxins and dibenzofurans
PCDF: Polychlorinated dibenzofuran
4/5-PL: Four/five-parameter logistic
pM: Picomolar concentration, pmol/L, 10^−12^ mol/L
REP: Relative potency (relative response of the CALUX cell system)
RLU: Relative light unit
RSD_Rw_: Relative standard deviation, within-laboratory reproducibility conditions
2,3,7,8-TCDD: 2,3,7,8,-Tetrachlorodibenzo-p-dioxin
TEF: Toxic equivalency factor
TEQ: Toxic equivalent
TWI: Tolerable weekly intake
SD: Standard deviation
SE: Standard error
U: Expanded measurement uncertainty
WHO: World Health Organisation
WSS: Weighted sum of squared residuals
WSSR: Weighted sum of squared residuals regression
XAP2: Hepatitis B virus X-associated protein 2
